# A Predictive Model and Risk Factors for Case Fatality of COVID-19

**DOI:** 10.3390/jpm11010036

**Published:** 2021-01-08

**Authors:** Melchor Álvarez-Mon, Miguel A. Ortega, Óscar Gasulla, Jordi Fortuny-Profitós, Ferran A. Mazaira-Font, Pablo Saurina, Jorge Monserrat, María N. Plana, Daniel Troncoso, José Sanz Moreno, Benjamin Muñoz, Alberto Arranz, Jose F. Varona, Alejandro Lopez-Escobar, Angel Asúnsolo-del Barco

**Affiliations:** 1 Service of Internal Medicine and Immune System Diseases-Rheumatology, University Hospital Príncipe de Asturias, (CIBEREHD), 28806 Alcalá de Henares, Spain; planafn@gmail.com (M.N.P.); jsanz.hupa@salud.madrid.org (J.S.M.); benmunozc@hotmail.com (B.M.); jarranzhupa@gmail.com (A.A.); 2 Department of Medicine and Medical Specialities, Faculty of Medicine and Health Sciences, University of Alcalá, 28801 Alcalá de Henares, Spain; miguel.angel.ortega92@gmail.com (M.A.O.); jorge.monserrat@uah.es (J.M.); daniel.troncoso@salud.madrid.org (D.T.); 3 Ramón y Cajal Institute of Sanitary Research (IRYCIS), 28034 Madrid, Spain; angel.asunsolo@uah.es; 4 Cancer Registry and Pathology Department, Hospital Universitario Principe de Asturias, 28806 Alcalá de Henares, Spain; 5 Hospital Universitari de Bellvitge–Universitat de Barcelona, 08907 L´Hospitalet de Llobregat, Spain; ogasulla@bellvitgehospital.cat; 6 Campus Nord, Universitat Politècnica de Catalunya, 08034 Barcelona, Spain; jordi.fortuny.profitos@estudiant.upc.edu; 7 Departament d’Econometria, Estadística I Economia Aplicada–Universitat de Barcelona, 08007 Barcelona, Spain; ferranmazaira@gmail.com; 8 Universidad Complutense de Madrid, 28040 Madrid, Spain; psaurina@ucm.es; 9 Service of Internal Medicine, Hospital Universitario HM Montepríncipe, HM Hospitales, 28660 Boadilla del Monte, Spain; jfvarona@hmhospitales.com; 10 Faculty of Medicine, Universidad San Pablo-CEU, CEU Universities, Fundación de Investigación HM Hospitales, 28003 Madrid, Spain; alopezescobar@hmhospitales.com; 11 Service of Pediatrics, Hospital Universitario HM Puerta del Sur, 28938 Móstoles, Spain; 12 Department of Surgery, Medical and Social Sciences, Faculty of Medicine and Health Sciences, University of Alcalá, 28801 Alcala de Henares, Spain; 13 Department of Epidemiology and Biostatistics, Graduate School of Public Health and Health Policy, University of New York, New York, NY 10027, USA

**Keywords:** COVID-19, C-reactive protein, oxygen saturation, ICU, death, predictive model

## Abstract

This study aimed to create an individualized analysis model of the risk of intensive care unit (ICU) admission or death for coronavirus disease 2019 (COVID-19) patients as a tool for the rapid clinical management of hospitalized patients in order to achieve a resilience of medical resources. This is an observational, analytical, retrospective cohort study with longitudinal follow-up. Data were collected from the medical records of 3489 patients diagnosed with COVID-19 using RT-qPCR in the period of highest community transmission recorded in Europe to date: February–June 2020. The study was carried out in in two health areas of hospital care in the Madrid region: the central area of the Madrid capital (Hospitales de Madrid del Grupo HM Hospitales (CH-HM), *n* = 1931) and the metropolitan area of Madrid (Hospital Universitario Príncipe de Asturias (MH-HUPA) *n* = 1558). By using a regression model, we observed how the different patient variables had unequal importance. Among all the analyzed variables, basal oxygen saturation was found to have the highest relative importance with a value of 20.3%, followed by age (17.7%), lymphocyte/leukocyte ratio (14.4%), CRP value (12.5%), comorbidities (12.5%), and leukocyte count (8.9%). Three levels of risk of ICU/death were established: low-risk level (<5%), medium-risk level (5–20%), and high-risk level (>20%). At the high-risk level, 13% needed ICU admission, 29% died, and 37% had an ICU–death outcome. This predictive model allowed us to individualize the risk for worse outcome for hospitalized patients affected by COVID-19.

## 1. Introduction

By the end of 2019, a novel coronavirus designated severe acute respiratory syndrome coronavirus 2 (SARS-CoV-2) was first reported in the city of Wuhan, the capital of Hubei, China, and caused an outbreak of unusual viral pneumonia [1]. Being highly transmissible, this novel coronavirus disease, also known as coronavirus disease 2019 (COVID-19), has spread rapidly all over the world and has posed an extraordinary threat to global public health [2]. Due to the high mortality rate and lack of optimal therapies, understanding the clinical features is crucial to responding to COVID-19. Rapid diagnoses and effective therapies are also important interventions for control the pandemic. The incidence, prevalence, and rapid evolution of SARS-CoV-2 infection are changing in all countries, and thus it is necessary to develop appropriate, dynamic, and transversal protocols to face the changing needs [3].

The clinical spectrum of SARS-CoV-2 infection appears to be wide, encompassing asymptomatic infection, mild upper respiratory tract illness, and severe viral pneumonia with respiratory failure and even death, with many patients being hospitalized with pneumonia [4,5,6,7]. The rapid growth in the number of cases, especially the critically ill or fatal patients, has posed a magnificent challenge on health systems, particularly in some countries, where there has been a sustained increase in COVID-19 cases during the last months [8]. Therefore, until an effective vaccine becomes widely available, there is an urgent need to decrease patient density, along with optimal clinical management of severe COVID-19 patients, finding new methods and approaches to achieve effective results even amidst this complex situation. This point would be of special importance, since it would allow better management of the resources at a time when a major economic recession has shaken up the world [9].

A strategic objective for clinical management of COVID-19 patients is to recognize critically ill patients in the early phases of the disease in order to adjust the treatment plan and assign medical resources rationally to improve the medical efficacy and reduce the risk of in hospital mortality. Several studies have analyzed the clinical and laboratory characteristics of critically ill COVID-19 patients [10]. These variables may provide a great support in establishing COVID-19 prognosis, helping to distinguish between non-severe and severe COVID-19 cases. Among these variables, serological, biochemical, immunological, and coagulation parameters have proven its remarkable importance in the stratification of COVID-19 progression [11,12]. Shen et al. [13] described the characteristic protein and metabolite changes in the sera of severe COVID-19 patients, which might be used in selection of potential blood biomarkers for severity evaluation. However, understanding and interpreting current available tests to recognize and hospitalize high-risk severe COVID-19 patients remains a challenge, generating uncertainty for patients, families, and healthcare professionals [14,15]. Rubio-Rivas et al. [15] have shown the existence of different phenotypes of patients on the basis of their comorbidities and their clinical characteristics, relating how it can be effective at the healthcare level in clinical management from the point of view of survival. Along these lines, other studies have unified symptoms and signs of manifestation of this disease with the aim of improving and implementing an action model [16,17,18].

Early identification of high-risk COVID-19 cases remains a crucial element in the clinical management of this disease. Therefore, establishing the prognostic score of COVID-19 patients requiring hospital admission is a relevant objective to optimize the clinical management of these patients. In our study, we analyzed the clinical characteristics of COVID-19 patients with worse outcome and non-critical illness, comparing their clinical and laboratory parameters to identify patient’s risk to develop the most severe presentation of COVID-19, helping physicians to focus on patients in greatest need.

## 2. Patients and Methods

### 2.1. Study Design and Patient Cohort

The present study was designed as an observational, analytical, retrospective cohort study with longitudinal follow-up. The conduct of the research and the dynamics of the study was carried out in accordance with the Strengthening of the Observational Studies Report in Epidemiology Guidelines (STROBE) [19].

This study was carried out with data collected from the medical records of 3489 patients diagnosed with COVID-19 according to the criteria established by the World Health Organization (WHO) by a real-time reverse transcription polymerase chain reaction (RT-qPCR) test taken from a nasopharyngeal sample [20]. The cohort was collected in the period of greatest community transmission recorded in Europe to date: February to June 2020. The study was carried out in parallel in two health areas of hospital care in the Madrid region: the central area of the capital of Madrid (Grupo HM-Hospitales de Madrid (CH-HM)) and the metropolitan area of Madrid (Hospital Universitario Príncipe de Asturias (MH-HUPA)). Of the total of 3489 patients, 1931 were evaluated at CH-HM and 1558 at MH-HUPA. The data were collected at the time of admission to the hospital, with the information in the electronic health records (EHR).

EHR consist of gender, age, comorbidities (Table 1), and results of basal oxygen saturation level and blood tests for white leukocytes and lymphocytes, C-reactive protein (CRP), and D-dimer at the hospital. Four-level categories were established that represent oxygen saturation (standard criteria):

1. Extremely low level corresponds to less than or equal to 80%.
2. Low level between 81% and 90%.
3. Medium between 91% and 94%.
4. Adequate to >94%.

As an alternative specification for the comorbidity score, we used the Charlson Index [21] and the Elixhauser Index [22]. For each patient, there is a dummy variable that indicates if a bed was provided in the intensive care unit (ICU) and if the patient died from COVID-19 (Table S1 presents the variables considered for the analysis).

### 2.2. Data Analysis

Data obtained from the study were included in a Microsoft Office Excel database (Microsoft, Redmond, Washington DC, USA) and R. Differences with *p* < 0.05 were considered statistically significant. Quantitative variables were expressed as mean (interquartile range or 95% confidence interval (CI)) and categorical variables as the number of patients and rates (%) (CI 95%). Univariate analysis was performed with Fisher’s test, χ^2^ test, and Student’s *t*-test, as appropriate.

To assess the risk of worse outcome (ICU or death) by COVID-19 at hospital admission, we followed a three-step process. First, we modeled the risk of worse outcome by specifying logistic regressions with different covariates, considering only data from the CH-HM (first cohort). Secondly, we validated the models trained with the CH-HM dataset by testing them to the MH-HUPA dataset (second cohort). Thirdly, we built final versions of the models using the whole dataset (total cohort). No imputations of the data were made.

We started with a basic model where the target was the dummy variable ICU or death and the covariates were variables previous to any medical test: age, gender, and comorbidities. We built a second model including oxygen saturation, which is the cheapest, fastest, and most readily available variable to obtain. Our third model included the standard indicators of a blood analysis: C-reactive protein (CRP), leukocyte level, lymphocyte/leukocyte ratio, and D-dimer. Finally, to gain a complete understanding of the model beyond the significance of the parameters, we estimated the relative importance of each variable included in the model, using a new methodology for model interpretation suggested by Lundeberg and Lee [23]: SHAP (SHapley Additive ExPlanation) values. On synthesis, given an observation $x=\left(x_{1},\ldots ,x_{J}\right)$, the SHAP value of feature *j* on instance *x* corresponds to the way in which the concrete value of feature *j* on *x* modifies the output of the model with respect to other instances that share some features with *x* but not *j*. For a parametric model $F\left(x\right)=g\left(\underset{j}{\sum }\alpha_{j}x_{j}\right)$, where g is a function of the weighted features of *x,* the SHAP value corresponds to $\varphi_{j}\left(x\right)=\alpha_{j}\left(x_{j}-E\left(X_{j}\right)\right)$, where *X* is the set of observations and $E\left(X_{j}\right)$ is the average value of the *j* feature on *X*. Then, noting as *N* the total number of observations, we can estimate the 164 relative importance of feature *j* in the model, RIj, as Equation (1)

(1) $$RI_{j}=\frac{\sum_{i=1}^{n}\left|\varphi_{j}\left(x_{i}\right)\right|}{\sum_{k=1}^{J}\sum_{i=1}^{n}\left|\varphi_{k}\left(x_{i}\right)\right|}$$

**Equation (1).** Formula for calculating the relative importance of the parameters to be analyzed, where the relative weight of each variable included in the model is obtained, using Shapley Additive ExPlanation (SHAP) values. Concretely, the relative importance of a variable is the sum of the absolute marginal contribution of the variable into the output of the model for all the observations (*i* = 1, …, N), divided by the sum of the absolute marginal contributions of all the variables (*k* = 1, …, J) for all the observations (*i* = 1, …, N).

### 2.3. Ethical Approval

This study was conducted according to basic ethical principles (autonomy, harmless, benefit, and distributive justice); its development followed the standards of Good Clinical Practice and the principles enunciated in the last Declaration of Helsinki (2013) and the Oviedo Convention (1997). The project was approved by the ethics committee of the University Hospital Príncipe de Asturias (HUPA-04062020).

## 3. Results

### 3.1. Patients Characteristics

We evaluated a total of 3489 patients, distributed in two health areas in the Madrid region. The mean age was 67.6 years, with 41.7% being female. The average total number of comorbidities was 3.3, with a Charlson Index of 0.9 and an Elixhauser Index of 2.0 (Table 2). The basal oxygen saturation level was extremely low in 4.4% and 15.7%. The highest percentage of patients (45.7%) maintained a basal oxygen saturation medium mean value of CRP of 74.3 µg/dL, mean leukocyte value of 7.8 10^3^/L, lymphocyte/leukocyte ratio of 18.7%, and mean D-dimer value of 2753.1 mg/L (Table 2). A total of 241 patients (6.9%) were admitted to ICU and 597 patients (17.3%) passed away. In total, 774 patients (22.1%) combined an episode of ICU admission or death (Table 2).

The mean age of the CH-HM patient group (*n* = 1931) was 68.4 years, and 41% were women. The presence of comorbidities was averaged 2.9 (Table 1 and Table 2). The Charlson Index and Elixhauser Index analysis for the CH-HM patients’ comorbidities were 0.7 and 1.8, respectively. Regarding basal oxygen saturation level, 4% patients were extremely low, 16.7% low, while the highest percentage of 30.1% was medium. Assessing analytical values in the CH-CM patient group, we found that the mean CRP was 73.8 µg/dL, leukocytes were 7.8 10^3^/L, lymphocyte/leukocyte ratio was 18.9%, and D-dimer was 2480.9 mg/L (Table 2). A total of 133 (6.9%) patients belonging this group were admitted to the ICU and 278 (14.4%) passed away. In total, 371 (19.2%) patients had combined ICU admission and death (Table 2).

With regard to the MH-HUPA patient group (*n* = 1558), the mean age was 66.7 years and 42.5% were female. The mean of comorbidities in this group was 3.8, and the Charlson Index and Elixhauser Index were 0.7 and 1.0, respectively. These patients showed a basal oxygen saturation that was extremely low at 4.9%, 14.3% was low, and 68.5% was medium. With regards to analytical data, the mean CRP was 75.0 µg/dL, mean leukocyte values were 73.3 10^3^/L, lymphocyte/leukocyte ratio was 18.6% and D-dimer was 3180.2 mg/L. A total of 109 (7%) patients were admitted to ICU, and 327 (20.9%) patients passed away, with 399 (25.6%) patients both admitted to ICU and having passed away (Table 2).

The average variance inflation factor (VIF) of the covariates for the CH-HM data was 1.19, and the maximum was 1.36. For the whole dataset (CH-HM and MH-HUPA), the average VIF was 1.20, and the maximum was 1.33. Hence, there was no evidence of a problem of multicolinearity.

### 3.2. Empirical Model and Results

To assess the risk of severe outcomes (ICU or *death*) of COVID-19 patients at hospital admission, we implemented a three-step process. Firstly, we built a model to analyze the importance of age, sex, and comorbidities using CH-HM patients (Table 3, EM-1). The parameters analyzed were statistically significant and the area under the curve was 0.7470. Secondly, the level of basal oxygen saturation was added to the model (Table 3, EM-2). Finally, analytical parameters (CRP, leukocytes, lymphocyte/leukocyte ratio, and D-dimer) were included. In the final model, all coefficients maintained their significance (Table 3, EM-3).

Additionally, we tested alternative specifications of comorbidities, such as the Charlson Index (Table 4, EM-4) or the Elixhauser Index (Table 4, EM-5). The comorbidity score adjusted for COVID-19 is slightly more informative that global scores.

### 3.3. Robustness Check

To validate the quality of the model for patients that were not included in the training set and to check that it was not biased by any particular medical procedure implemented at the CH-HM, we tested the constructed model in 1558 patients from the MH-HUPA. The accuracy of fit of the model when applied to the MH-HUPA patients was almost the same as when it was applied to the CH-HM. Hence, the model was shown to be robust to different hospitals and did not present any bias, as in all cases the AUC was greater than 0.7 and the difference in the AUC between the two cohorts was lower than 2.2 percentage points (Table 5).

### 3.4. Final Models and Interpretation

Once the initial models were validated, we ran the models using the complete cohort including patients from both groups, namely, the CH-HM and the MH-HUPA. Estimated coefficients, levels of significance, and AUC were almost identical with respect to the CH-CM dataset (Table 6).

Finally, to gain a complete understanding of the model beyond the significance of the parameters, we estimated the relative weight of each variable included in the model using SHAP values Equation (1). Table 7 represents the relative importance of each variable in the estimates (6) to (8). The highest relative importance for the complete model (EM-8) was for basal oxygen saturation (20.3%), followed by age (17.7%), lymphocyte/leukocyte ratio (14.4%), and CRP analysis and comorbidities (12.5% each). Smaller values of relative importance were observed in leukocytes (8.9%), female gender (6.9%), and D-dimer levels (6.7%).

### 3.5. Categories of Individualized Risk

Our results establish a clinical score that allows to us estimate the individualized risk for severe outcomes in COVID-19 hospitalized patients. Supplementary material examples of this application are included (Table S2).

**Figure 1:**
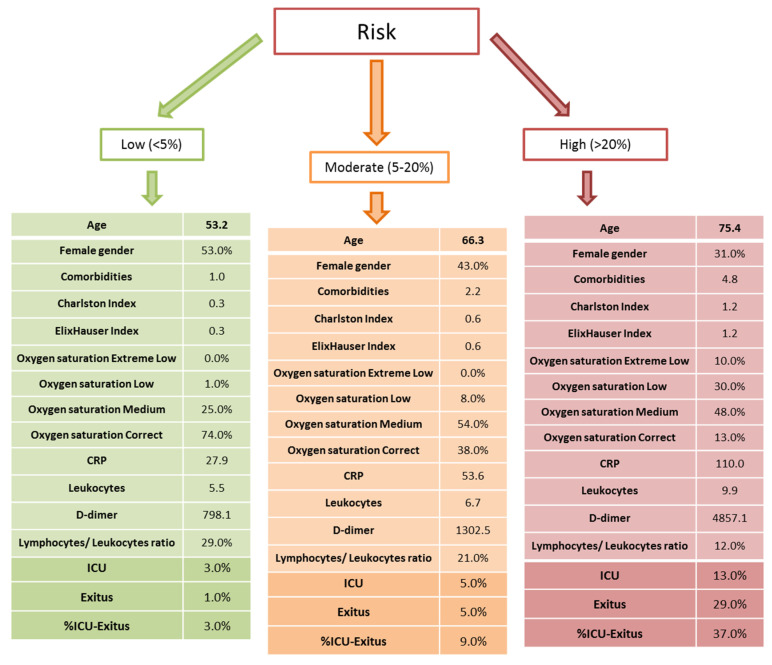
Graphic representation to classify the importance of the study variables in relation to admission to the ICU and *death*. Three levels of risk are described in this model: low (<5%), moderate (5–20%), and high (>20%). The results are expressed as mean (standard deviation). CRP = C-reactive protein mean, ICU = intensive care unit.

Furthermore, the model allows us to categorize the risk of ICU or in-hospital mortality in three risk categories (Figure 1). The low-risk category (<5%), by our definition, contained 554 patients with a mean age of 53.2 years, with 53.0% being female. At this low-risk level, the mean level of comorbidities was 1.0 with the Charlson Index and Elixhauser Index, and 0.3 in both. It is important to note how the highest percentage of patients (74.0%) had adequate basal oxygen saturation. The analytical parameters were 27.9 for CRP, 5.5 for leukocytes, 29.0% for lymphocyte/leukocyte ratio and 798.1 for D-dimer. At this low-risk level, only 3% patients had to be admitted to ICU, 1.0% *died*, and 3% had a ICU–*death* outcome (Figure 1).

In a second point, we identified a medium-risk category (5–20%) for ICU admission or in hospital mortality that included 933 patients with a mean age of 66.3 years, with 43.0% being female. Within this medium-risk level, we observed comorbidities levels of 2.2 with the Charlson Index and Elixhauser Index, and 0.6 for the two indexes. A total of 54.0% of the patients had a mean level of basal oxygen saturation, and analytical parameters were 53.6 for CRP, 6.7 for leukocytes, 21.0% for lymphocyte/leukocyte ratio, and 1302.5 for D-dimer. At this medium-risk level, only 5.0% needed to be admitted to ICU, 5.0% died, and 9.0% had an ICU–death outcome (Figure 1).

Finally, we identified a high-risk category (>20%) that included 1177 patients. The mean age in this group was 75.4 years, with 31.0% being female. At this high-risk level, the mean levels of comorbidities were the highest, with 4.8 for the Charlston Index and an Elixhauser Index of 1.2. It is important to note that the highest percentage of patients (48.0%) had basal oxygen saturation at medium levels and only 13.0% was adequate. The analytical parameters were 110.0 for CRP, 9.9 for leukocytes, 37.0% for lymphocyte/leukocyte ratio, and 4857.1 for D-dimer. At this high-risk level, 13% were admitted to ICU, 29.0% *died*, and 37% had an ICU–death outcome (Figure 1).

## 4. Discussion

According to our results, clinical and analytical parameters can be used to identify patients at high risk of severe forms of COVID-19. The use of predictive models was proven useful in the detection of high-risk patients, which may be a good tool for implementing rapid response interventions, reducing mortality [24]. At present, numerous authors have described how, in large cohorts, it is possible to describe a series of standards that allow clinical decision-making [17]. In the present study, we show a predictive model in patients with COVID-19 in order to identify determinants of ICU admission and death.

In our regression model, the basal oxygen saturation level was found to be the most important predictive clinical marker to detect cases in which additional support is needed due to their higher risk of death. This was followed by the serum levels of CRP, age, the presence of comorbidities, leukocytes, and lymphocyte/leukocyte ratio. Our results are partly consistent with previous studies such as Allenbach et al. [25], who described that older age, respiratory insufficiency, higher CRP levels, and lower lymphocytes in blood are strongly associated with an increased risk of ICU admission and mortality. Similarly, Xie et al. [26] conducted a multivariable model on admission reporting oxygen saturation, age, lymphocyte count, and lactate dehydrogenase as independent predictors of mortality.

A wide range of studies have unraveled the important use of oxygen saturation as a predictive value of severity [27] or hospitalization [28]. Likewise, monitoring oxygen saturation is an excellent way to reduce bed demands, thus allowing a more efficient management of the available resources [29]. Hypoxemic respiratory failure is the main cause of ICU admission [30]. Consistent with this study, Zhao et al. [31] reported that oxygen saturation could be used either as a significant variable predicting ICU admission or as a predictor of mortality in patients with COVID-19. Our study shows an up to 20.3% association among this parameter and prognosis in patients with COVID-19, being the most important factor to consider in the prediction of both admission in the ICU and its later mortality.

CRP is an inflammatory marker easily and commonly measured, which appears to be increased in COVID-19 patients. Related to disease severity, it has been suggested to represent a prognosis marker in several studies, and also to evaluate ICU admission [32]. For example, concentration of 100 mg/L of CRP has been described to implicate increased risk of ICU, prolonged length of stay, and an increase at one-month mortality [33]; in other observational studies, serum levels of CRP allowed the researchers to recognize alarming cases of COVID-19 [34]. CRP also correlates with decreased levels of red blood cells, neutrophil count, and lymphocytes in the early stages of the disease [32,35]. Increased CRP levels may reflect diameter of lung lesion as well, as both variables have been shown to correlate positively [36]. Immunological parameters including higher leucocytes and reduced lymphocytes directly correlate with severity and worse prognosis in ICU patients [37]. Our study described lymphocyte/leucocyte ratio (14.4%) and leucocytes (8.9%) as major predictive factors of both ICU admission and mortality, thereby denoting the relevance of immunological markers in COVID-19 patients.

Furthermore, our research also considered age (17.7%), comorbidities (12.5%), female gender (6.9%), and D-dimer value (6.7%) as independent variables of ICU admission and mortality. In this context, Larsson et al. [38] showed the central role of age (>59 years old) in ICU admission and mortality. In estimations EM-1 to EM-3, age is associated, in a quadratic form, with a higher risk of fatal outcome. Females are more resistant to COVID-19 than men, as reported by [39,40], among others. Previous comorbidities are also associated with a higher risk of mortality; these data are consistent with the reported information of numerous studies [41], as well as low oxygen saturation levels; high levels of CRP, leukocytes, and D-dimer; and a low lymphocyte/leukocyte ratio [27,42].

Haase et al. [43] identified age, male gender, and different comorbidities such as chronic pulmonary disease (COPD) with increased risk of death in 323 patients admitted to ICUs. Consistent with these results, Zhao et al. [31] also reported age and COPD as major variables predicting mortality. Likewise, type 2 diabetes mellitus has also been postulated as a direct risk factor for ICU admission and mortality of COVID-19, being the second most common comorbidity in these patients [44]. According to our study, the most part of hospitalized patients are male [45]. Contrary to our study, this has also been found to be associated with higher risk of mortality [46]. Notwithstanding, the real impact of sex in COVID-19 admission and mortality remains to be elucidated as there is some discordance in the different data [47]. We show that female gender is an important factor to consider in the ICU admission and as a prognostic factor in patients with COVID-19. Coagulation disorders is a common problem of COVID-19, with an approximately incidence of one in three patients [48]. D-dimer is a variable that appears to be elevated in >95% ICU patients [49]. Thus, D-dimer effectively predicts, in other studies, in-hospital mortality, showing its potential in early prognosis in patients with COVID-19 [50]. Our study expressed a significant correlation between this parameter with mortality and admission in ICU, which may provide a helpful support for clinicians in the management of these patients.

Recently, different studies have attempted to classify patients by phenotypes [18]. Our study allowed us to obtain risk levels regardless of specific phenotypes of patients, only taking into account rapid clinical parameters that allow decision-making in an effective way to save patients´ lives.

The reduced numbed of analytical variables of the patients included in this study may be considered a limitation. The relevance of genetic and biological factors for the susceptibility and evolution of COVID-19 have been described. There is increasing evidence that deficiency or overexpression of various immunological molecules and cellular populations as well as different genetic factors condition the clinical evolution and severity COVID-19 [51,52,53]. The inclusion of biological parameters of the immune-inflammatory and neuro-endocrine metabolic response of the patient to SARS-CoV-2 infection may improve the understanding of the pathogenesis of the disease and the clinical and therapeutic management of COVID-19 patients. However, the aim of our study was to develop a predictive and individualized model of risk for worse outcome for hospitalized patients by COVID-19. The described model allows for a quick and effective decision-making process for COVID-19 patients in the medical emergency rooms and at hospital admission. This is an important point and that can be considered as one of the limitations of our study. However, for the first time, we managed to develop a rapid risk model for medical emergencies.

## 5. Conclusions

Our results show a model that allows for prediction of the level of risk of a patient with a diagnosis of COVID-19 (by PCR) of suffering an ICU admission. It provides the advantage of considering simple covariates prior to any medical examination, such as age, sex, and comorbidities, together with rapidly accessible quantitative parameters in the hospital emergency (basal oxygen saturation and CRP levels). Our model estimated that 37.0% of patients classified as high-risk are admitted to ICU and die.

## Figures and Tables

**Table 1:** Description of the comborbidities of the coronavirus disease 2019 (COVID-19) patients included in the study. Results include those of the Grupo Hospitales de Madrid (CH-HM) at the central area of Madrid, and those of the Hospital Universitario Príncipe de Asturias (MH-HUPA) at the metropolitan area of Madrid. We also present the results of the two cohorts together (total cohort). For each comorbidity, we present the percentage of patients (with respect to the group they belong to) suffering from the comorbidity, and the odds ratio of death (OR) for the indicated comorbidity in the studied population. The weight each comorbidity is given in the comorbidity score is estimated as a weighted average between the odds ratio of the disease (in the training set, which is the CH-HM cohort for the estimates EM-1 to EM-5, and the Global for the EM-6 to EM-8) and the odds ratio of the comorbidity group, which is included as a Bayesian prior of 30 patients. Hence, for a comorbidity C diagnosed to 100 patients, from group G, the weight w given as $w=\frac{100\;OR^{C}+30\;OR^{G}}{130}$, where $OR^{C}$ is the odds ratio of the disease and $OR^{G}$ is that of the group. In the table, we present the weights for the CH-HM cohort. For example, for COR pulmonale, the weight is $w=\frac{100\;*\;2.37\;+30\;*\;2.21}{130}=2.33$.

		CH-HM		MH-HUPA		Total		
Group	Comorbidities	%	OR	%	OR	%	OR	Weight
Hypertension	Arterial hypertension	42.5%	1.90	65.6%	1.77	45.6%	1.90	1.90
	Total	42.5%	1.90	65.6%	1.77	45.6%	1.90	-
Cardiopathies	COR pulmonale	6.8%	2.37	9.8%	1.67	7.3%	2.14	2.33
	Ischemic heart disease	6.9%	1.69	10.4%	1.72	7.1%	1.77	1.81
	Heart failure	4.8%	2.13	10.8%	3.54	6.6%	2.98	2.15
	Total	15.8%	2.21	25.5%	2.48	17.5%	2.44	-
Metabolic–endocrine	Diabetes	17.3%	1.44	30.6%	1.48	20.1%	1.53	1.39
	Hypothyroidism	6.8%	0.74	6.8%	0.73	6.3%	0.70	0.85
	Obesity	7.5%	1.16	13.5%	1.23	8.8%	1.35	1.17
	Total	28.1%	1.21	42.6%	1.30	30.3%	1.31	-
Respiratory illnesses	ASTHMA	4.7%	0.91	11.6%	0.68	6.4%	0.78	1.05
	COPD	7.8%	2.20	12.2%	1.79	7.9%	2.18	2.05
	Others	2.1%	1.37	4.7%	1.67	2.6%	1.56	1.41
	Total	13.7%	1.55	24.5%	1.25	15.3%	1.46	-
Nephropathies	Acute renal failure	8.9%	6.36	16.9%	3.04	11.5%	4.64	6.03
	Chronic renal failure	5.6%	2.22	13.7%	2.20	7.3%	2.50	2.85
	Total	11.8%	4.97	25.0%	2.66	15.1%	3.91	-
Solid neoplasia	Colon cancer	0.7%	6.85	0.7%	2.53	0.6%	5.36	6.01
	Breast cancer	0.5%	1.81	0.3%	2.02	0.4%	1.57	2.14
	Respiratory/lung cancer	1.2%	2.56	1.5%	0.79	1.0%	1.86	2.72
	Total	2.4%	3.24	2.6%	1.30	1.9%	2.53	-
Hematological neoplasia	Leukemia	0.9%	1.63	1.4%	2.55	0.9%	2.06	1.65
	Lymphoma	0.8%	1.92	1.0%	0.46	0.6%	1.65	1.88
	Multiple myeloma	0.6%	1.58	0.7%	1.73	0.6%	1.52	1.62
	Total	2.3%	1.73	3.1%	1.53	2.1%	1.79	-
Autoimmune intestinalis	Ulcerative colitis	0.5%	1.81	0.4%	1.21	0.5%	1.47	1.81
	Total	0.5%	1.81	0.4%	1.21	0.5%	1.47	-
Autoimmune rheumatological	Rheumatoid arthritis	1.1%	0.66	2.1%	1.52	1.4%	1.28	0.81
	Spondyloarthritis	3.5%	1.45	3.0%	1.63	3.0%	1.28	1.42
	Psoriasis	0.8%	1.05	2.4%	0.47	1.0%	0.70	1.12
	Vasculitis	1.5%	1.70	4.3%	1.20	2.5%	1.28	1.61
	Total	6.6%	1.33	11.6%	1.22	7.7%	1.23	-
Urinary infection	Urinary infection	4.6%	2.04	7.2%	1.46	5.3%	1.85	2.04
	Total	4.6%	2.04	7.2%	1.46	5.3%	1.85	-
Neurological	Dementia	3.7%	1.94	4.0%	1.05	3.5%	1.52	1.94
	Total	3.7%	1.94	4.0%	1.05	3.5%	1.52	-

**Table 2:** Description of the COVID-19 patients included in the study. Results include those of the Grupo Hospitales de Madrid (CH-HM) at the central area of Madrid, and those of the Hospital Universitario Príncipe de Asturias (MH-HUPA) at the metropolitan area of Madrid. We also present the results of the two cohorts together (total cohort). Global description of the patients included in the study showing the mean (standard deviation) values of the demographic, clinical, and analytical characteristics of the patients, as well as the percentage of admissions to the intensive care unit (ICU) and deaths. The results are expressed as mean (standard deviation). CRP = C-reactive protein, *n* = number.

	CH-HM (*n* = 1931)	MH-HUPA (*n* = 1558)	Total (*n* = 3489)
Age	68.4 (16.3)	66.7 (16.2)	67.6 (16.3)
Female gender	41.0%	42.5%	41.7%
Comorbidities	2.9 (3.4)	3.8 (4.3)	3.3 (3.9)
Charlson Index	0.7 (1.0)	1.0 (1.3)	0.9 (1.2)
Elixhauser Index	1.8 (1.7)	2.2 (2.2)	2.0 (1.9)
Oxygen saturation extremely low	4.0%	4.9%	4.4%
Oxygen saturation low	16.7%	14.3%	15.7%
Oxygen saturation medium	30.1%	68.5%	45.7%
Oxygen saturation correct	49.1%	12.2%	34.2%
CRP (μg/dL)	73.8 (85.3)	75.0 (80.6)	74.3 (83.4)
Leukocytes (10^3^/L)	7.8 (4.9)	7.8 (5.0)	7.8 (4.9)
D-dimer (mg/L)	2480.9 (6952.8)	3180.2 (14,092.7)	2753.1 (10,338.2)
Lymphocyte/leukocyte ratio	18.9% (11.0%)	18.5% (11.0%)	18.7% (11.2%)
ICU	6.9%	7.0%	6.9%
Death	14.4%	20.9%	17.3%
%ICU or death	19.2%	25.6%	22.1%
%ICU and death	2.1%	2.3%	2.1%

**Table 3:** Estimated parameters of the models in Grupo Hospitales de Madrid (CH-HM) dataset. Standard errors in brackets. AUC = area under the curve.

	EM-1	*p*-Value	EM-2	*p*-Value	EM-3 ^a^	*p*-Value
Age	0.0394 ***	0.0000	0.0364 ***	0.0000	0.0299 ***	0.0000
	(0.0050)		(0.0052)		(0.0061)	
Female gender	−0.5482 ***	0.0000	−0.4613 ***	0.0007	−0.3935 *	0.0159
	(0.1313)		(0.1369)		(0.1632)	
Comorbidities	0.1190 ***	0.0000	0.01253 ***	0.0000	0.1233 ***	0.0000
	(0.0175)		(0.0179)		(0.0210)	
Oxygen saturation extremely low	-	-	2.4175 ***	0.0000	1.7599 ***	0.0000
	-		(0.2680)		(0.3074)	
Oxygen saturation low	-	-	1.1536 ***	0.0000	0.7813 ***	0.0001
	-		(0.1666)		(0.2021)	
Oxygen saturation medium	-	-	0.6068 ***	0.0000	0.5772 **	0.0016
			(0.1561)		(0.1826)	
CRP (μg/dL)	-	-	-	-	0.0031 ***	0.0001
	-		-		(0.0008)	
Leukocytes (10^3^/L)	-	-	-	-	0.0596 ***	0.0001
	-		-		(0.0154)	
Lymphocyte/leukocyte ratio	-	-	-	-	−3.8257 ***	0.0000
	-		-		(0.8623)	
D-dimer/Reference value ^1^	-	-	-	-	0.0149 **	0.0011
	-		-		(0.0046)	
**No. observations**	**1.931**	**-**	**1.931**	**-**	**1.622**	**-**
**AUC**	**0.7470**	**-**	**0.7845**	**-**	**0.8187**	**-**

^a^ A total of 309 patients with missing blood indicators were excluded from EM-3. The Chow stability test on whether parameters in EM-1 and EM-2 are sensitive to patients with missing information does not reject the null hypothesis that are not sensitive (*p*-values > 0.25). Hence, the exclusion of these patients does not affect parameters in EM-3. ^1^ Reference value = 500 ng/Ml. Significance: *** *p* < 0.001, ** *p* < 0.01, * *p* < 0.05.

**Table 4:** Estimated parameters of the models in Grupo Hospitales de Madrid (CH-HM) dataset with alternative specifications for comorbidities.

	EM-3	*p*-Value	EM-4	*p*-Value	EM-5	*p*-Value
Age	0.0299 ***	0.0000	−0.0386 ***	0.0000	−0.0342 ***	0.0000
	(0.0061)		(0.0037)		(0.0060)	
Female gender	−0.3935 *	0.0159	−0.4541 **	0.0048	−0.4593 **	0.0044
	(0.1632)		(0.1611)		(0.1937)	
Comorbidities	0.1233 ***	0.0000	-	-	-	-
	(0.0210)		-		-	
Charlson Index	-	-	0.1898 **	0.0064	-	
	-		(0.0696)		-	
Elixhauser Index	-	-	-	-	0.1937 ***	0.0000
	-		-		(0.0451)	
Oxygen saturation extremely low	1.7599 ***	0.0000	1.7108 ***	0.0000	1.6997 ***	0.0000
	(0.3074)		(0.3058)		(0.3048)	
Oxygen saturation low	0.7813 ***	0.0001	0.8157 ***	0.0000	0.8020 **	0.0001
	(0.2021)		(0.1995)		(0.1998)	
Oxygen saturation medium	0.5772 **	0.0016	0.5751 **	0.0014	0.5818 **	0.0013
	(0.1826)		(0.1805)		(0.1813)	
CRP (μg/dL)	0.0031 ***	0.0001	0.0028 ***	0.0002	0.0031 ***	0.0001
	(0.0008)		(0.0008)		(0.0008)	
Leukocytes (10^3^/L)	0.0596 ***	0.0001	0.0612 ***	0.0000	0.0623 ***	0.0000
	(0.0154)		(0.0151)		(0.0153)	
Lymphocyte/leukocyte ratio	−3.8257 ***	0.0000	−3.9553 ***	0.0000	−3.8329 ***	0.0000
	(0.8623)		(0.8534)		(0.8623)	
D-dimer/Reference value ^1^	0.0149 **	0.0011	0.0155 ***	0.0009	0.0149 **	0.0013
	(0.0046)		(0.0047)		(0.0046)	
**No. Observations**	**1.622**	**-**	**1.622**	**-**	**1.622**	**-**
**AUC**	**0.8187**	**-**	**0.8059**	**-**	**0.8107**	**-**

^1^ Reference value = 500 ng/Ml. Standard errors in brackets. AUC = area under the curve. Significance: *** *p* < 0.001, ** *p* < 0.01, * *p* < 0.05.

**Table 5:** Estimated parameters of the models in Grupo Hospitales de Madrid (CH-HM) and Hospital Universitario Príncipe de Asturias (MH-HUPA) datasets.

	EM-1	EM-2	EM-3
AUC CH-HM	0.7494	0.7862	0.8247
AUC MH-HUPA	0.7273	0.7773	0.8076

AUC = area under the curve. EM-1 = empirical model 1, EM-2 = empirical model 2, EM-3 = empirical model 3.

**Table 6:** Estimated parameters of the models in Grupo Hospitales de Madrid (CH-HM) and Hospital Universitario Príncipe de Asturias (MH-HUPA) datasets with alternative specifications for comorbidities.

	EM-6	*p*-Value	EM-7	*p*-Value	EM-8	*p*-Value
Age	0.0417 ***	0.0000	0.0393 ***	0.0000	0.0254 ***	0.0000
	(0.0035)		(0.0038)		(0.0044)	
Female gender	−0.4843 ***	0.0000	−3762 ***	0.0001	−0.2733 *	0.0229
	(0.0912)		(0.1369)		(0.1632)	
Comorbidities	0.0964 ***	0.0000	0.0888 ***	0.0000	0.0830 ***	0.0000
	(0.0122)		(0.0132)		(0.0140)	
Oxygen saturation extremely low	-	-	3.1240 ***	0.0000	2.4006 ***	0.0000
	-		(0.2255)		(0.2571)	
Oxygen saturation low	-	-	1.4683 ***	0.0000	1.0468 ***	0.0000
	-		(0.1400)		(0.1687)	
Oxygen saturation medium	-	-	0.7976 ***	0.0000	0.6528 ***	0.0000
			(0.1218)		(0.1474)	
CRP (μg/dL)	-	-	-	-	0.0039 ***	0.0000
	-		-		(0.0006)	
Leukocytes (10^3^/L)	-	-	-	-	0.0562 ***	0.0000
	-		-		(0.0105)	
Lymphocyte/leukocyte ratio	-	-	-	-	−3.1445 ***	0.0000
	-		-		(0.6027)	
D-dimer/Reference value ^1^	-	-	-	-	0.0206 ***	0.0000
	-		-		(0.0041)	
**No. Observations**	**3.489**	**-**	**3.247**	**-**	**2.664**	**-**
**AUC**	**0.7317**	**-**	**0.7841**	**-**	**0.8177**	**-**

EM-6 = empirical model 6, EM-7 = empirical model 7, EM-8 = empirical model 8. ^1.^ Reference value = 500 ng/Ml. Standard errors in brackets. N = number of patients, AUC= area under the curve. Significance: *** *p* < 0.001, * *p* < 0.05.

**Table 7:** Relative importance of the variables in the total cohort of COVID-19 patients analyzed using a new methodology for model interpretation suggested by Lundeberg and Lee [16,17]: SHAP (SHapley Additive ExPlanation) values.

	EM-6	EM-7	EM-8
Age	50.9%	36.2%	17.7%
Female gender	24.4%	12.6%	6.9%
Comorbidities	24.7%	16.5%	12.5%
Oxygen saturation	-	34.6%	20.3%
CRP (μg/dL)	-	-	12.5%
Leukocytes (10^3^/L)	-	-	8.9%
Lymphocyte/leukocyte ratio	-	-	14.4%
D-dimer (mg/L)	-	-	6.7%

CRP = C-reactive protein mean. EM-6 = empirical model 6, EM-7 = empirical model 7, EM-8 = empirical model 8.

## Data Availability

The data presented in this study are available on request from the corresponding author.

## Supplementary Materials

The following are available online at https://www.mdpi.com/2075-4426/11/1/36/s1. Table S1: presents the variables considered for the analysis. Table S2: presents examples of this application.

## Abbreviations

COVID-19	Coronavirus disease 2019
RT-qPCR	Real-time reverse transcription polymerase chain reaction
ICU	Intensive care unit
CRP	C-reactive protein
