# Supplementary material for: A Predictive Model and Risk Factors for Case Fatality of COVID-19
Source: J Pers Med. 2021 Jan 8;11(1):36. doi: 10.3390/jpm11010036 (PMC7827846; doi:10.3390/jpm11010036)
Supplement: Supplementary file 1 [file jpm-11-00036-s001.zip › jpm-1051672-supplementary1.pdf]

	Date	Basic		Satu
	**Date**	Model	Aplication	Model
Constante		-4.3906	(4.3906)	-5.0732
Age (years)	70	0.0417	2.9184	0.0393
Female gender (1 yes/0 not)	0	-0.4843	-	-0.3762
Comorbidities	6.15	0.0964	0.5929	0.0888
Oxygen saturation Extreme Low	0			3.1240
Oxygen saturation Low	0			1.4683
Oxygen saturation Medium	1			0.7976
CRP (Proteína C-reactiva)	120			
Leukocytes	10.5			
Lymphocytes/ Leukocytes ratio	0.1			
D-Dimero	3.0			
**Score**			**(0.8794)**	
**Probability ICU-Exitus**			**29.3%**	

ration	Saturation and Analitic	
Aplication	Model	Aplication
(5.0732)	-4.4889	(4.4889)
2.7526	0.0254	1.7756
-	-0.2733	-
0.5460	0.0830	0.5102
-	2.4006	-
-	1.0468	-
0.7976	0.6528	0.6528
	0.0039	0.4642
	0.0562	0.5896
	-3.1445	(0.3145)
	0.0206	0.0617
**(0.9770)**		**(0.7491)**
**27.3%**		**32.1%**
