# Supplementary material for: A Predictive Model and Risk Factors for Case Fatality of COVID-19
Source: J Pers Med. 2021 Jan 8;11(1):36. doi: 10.3390/jpm11010036 (PMC7827846; doi:10.3390/jpm11010036)
Supplement: Supplementary file 1 [file jpm-11-00036-s001.zip › jpm-1051672-supplementary2.pdf]

		CH-HM		MH-HUPA		GLO
**Group**	**Comorbidities**	**%**	**OR**	**%**	**OR**	**%**
Hypertension	Arterial hypertension	42,5%	1,90	65,6%	1,77	45,6%
	Total	*42,5%*	*1,90*	*65,6%*	*1,77*	*45,6%*
Cardiopathies	COR pulmonale	6,8%	2,37	9,8%	1,67	7,3%
	Ischemic heart disease	6,9%	1,69	10,4%	1,72	7,1%
	Heart failure	4,8%	2,13	10,8%	3,54	6,6%
	Total	*15,8%*	*2,21*	*25,5%*	*2,48*	*17,5%*
Metabolic-endocrine	Diabetes	17,3%	1,44	30,6%	1,48	20,1%
	Hypothyroidism	6,8%	0,74	6,8%	0,73	6,3%
	Obesity	7,5%	1,16	13,5%	1,23	8,8%
	Total	*28,1%*	*1,21*	*42,6%*	*1,30*	*30,3%*
Respiratory illnesses	ASTHMA	4,7%	0,91	11,6%	0,68	6,4%
	COPD	7,8%	2,20	12,2%	1,79	7,9%
	Others	2,1%	1,37	4,7%	1,67	2,6%
	Total	*13,7%*	*1,55*	*24,5%*	*1,25*	*15,3%*
Nephropathies	Acute renal failure	8,9%	6,36	16,9%	3,04	11,5%
	Chronic renal failure	5,6%	2,22	13,7%	2,20	7,3%
	Total	*11,8%*	*4,97*	*25,0%*	*2,66*	*15,1%*
Solid neoplasia	Colon cancer	0,7%	6,85	0,7%	2,53	0,6%
	Breast cancer	0,5%	1,81	0,3%	2,02	0,4%
	Respiratory / lung cance	1,2%	2,56	1,5%	0,79	1,0%
	Total	*2,4%*	*3,24*	*2,6%*	*1,30*	*1,9%*
Hematological neoplasia	Leukemia	0,9%	1,63	1,4%	2,55	0,9%
	Lymphoma	0,8%	1,92	1,0%	0,46	0,6%
	Multiple myeloma	0,6%	1,58	0,7%	1,73	0,6%
	Total	*2,3%*	*1,73*	*3,1%*	*1,53*	*2,1%*
Autoimmune intestinalis	Ulcerative colitis	0,5%	1,81	0,4%	1,21	0,5%
	Total	*0,5%*	*1,81*	*0,4%*	*1,21*	*0,5%*
Autoimmune rheumatological	Rheumatoid arthritis	1,1%	0,66	2,1%	1,52	1,4%
	Spondyloarthritis	3,5%	1,45	3,0%	1,63	3,0%
	Psoriasis	0,8%	1,05	2,4%	0,47	1,0%
	Vasculitis	1,5%	1,70	4,3%	1,20	2,5%
	Total	*6,6%*	*1,33*	*11,6%*	*1,22*	*7,7%*
Urinary infection	Urinary infection	4,6%	2,04	7,2%	1,46	5,3%
	Total	*4,6%*	*2,04*	*7,2%*	*1,46*	*5,3%*
Neurological	Dementia	3,7%	1,94	4,0%	1,05	3,5%
	Total	*3,7%*	*1,94*	*4,0%*	*1,05*	*3,5%*

BAL		Present
OR	Weight	in patient
1,90	1.9	
1,90		
2,14	1.79	
1,77	2.34	
2,98	2.15	
2,44		
1,53	1.42	0
0,70	0.83	
1,35	1.17	
1,31		
0,78	1.07	
2,18	2.09	
1,56	1.45	
1,46		
4,64	6.15	1
2,50	2.82	
3,91		
5,36	4.33	0
1,57	2.88	
1,86	2.94	
2,53		
2,06	1.69	
1,65	1.8	
1,52	1.69	
1,79		
1,47	1.81	
1,47		
1,28	1.05	
1,28	1.41	
0,70	1.24	
1,28	1.51	
1,23		
1,85	2.04	
1,85		
1,52	1.94	
1,52		

**Total**	**6.15**
